# External Validation and Comparison of Three Pediatric Clinical Dehydration Scales

**DOI:** 10.1371/journal.pone.0095739

**Published:** 2014-05-02

**Authors:** Joshua Jauregui, Daniel Nelson, Esther Choo, Branden Stearns, Adam C. Levine, Otto Liebmann, Sachita P. Shah

**Affiliations:** 1 Warren Alpert Medical School Department of Emergency Medicine, Brown University, Providence, Rhode Island, United States of America; 2 Division of Emergency Medicine, University of Washington, Seattle, Washington, United States of America; 3 Rhode Island Hospital, Providence, Rhode Island, United States of America; University of Alabama at Birmingham, United States of America

## Abstract

**Objective:**

To prospectively validate three popular clinical dehydration scales and overall physician gestalt in children with vomiting or diarrhea relative to the criterion standard of percent weight change with rehydration.

**Methods:**

We prospectively enrolled a non-consecutive cohort of children ≤ 18 years of age with an acute episode of diarrhea or vomiting. Patient weight, clinical scale variables and physician clinical impression, or gestalt, were recorded before and after fluid resuscitation in the emergency department and upon hospital discharge. The percent weight change from presentation to discharge was used to calculate the degree of dehydration, with a weight change of ≥ 5% considered significant dehydration. Receiver operating characteristics (ROC) curves were constructed for each of the three clinical scales and physician gestalt. Sensitivity and specificity were calculated based on the best cut-points of the ROC curve.

**Results:**

We approached 209 patients, and of those, 148 were enrolled and 113 patients had complete data for analysis. Of these, 10.6% had significant dehydration based on our criterion standard. The Clinical Dehydration Scale (CDS) and Gorelick scales both had an area under the ROC curve (AUC) statistically different from the reference line with AUCs of 0.72 (95% CI 0.60, 0.84) and 0.71 (95% CI 0.57, 0.85) respectively. The World Health Organization (WHO) scale and physician gestalt had AUCs of 0.61 (95% CI 0.45, 0.77) and 0.61 (0.44, 0.78) respectively, which were not statistically significant.

**Conclusion:**

The Gorelick scale and Clinical Dehydration Scale were fair predictors of dehydration in children with diarrhea or vomiting. The World Health Organization scale and physician gestalt were not helpful predictors of dehydration in our cohort.

## Introduction

Dehydration remains a significant cause of morbidity and mortality in the pediatric population worldwide [1]. In the United States, gastroenteritis alone accounts for more than 1.5 millions outpatient visits, 200,000 admissions and 300 deaths annually [1]. Accurate assessment of the degree of dehydration can help clinicians guide treatment with either oral or intravenous fluid resuscitation, and is necessary for accurate prognosis and resource management [1]-[4]. In the literature, the established criterion standard for determining the degree of dehydration is retrospectively determined by the percent weight change before and after volume resuscitation [5]-[6]. However, this is not useful in the emergency department or acute care setting [7]. Clinically, the degree of dehydration in children is usually estimated based on historical and physical findings that lack a high degree of sensitivity, specificity and reliability [1].

Several studies have found that a combination of certain clinical signs and symptoms may better predict pediatric dehydration status than individual clinical characteristics [8]-[13]. Prior research conducted at the Hospital for Sick Children in Toronto resulted in the creation of the Clinical Dehydration Scale (CDS) for use in children from 1 month to 3 years of age [11]. Other scales in use for children 1 month to 5 years of age have been developed by the World Health Organization (WHO) and Gorelick et. al. and are displayed in Tables 1-3 [10], [12]. These popular scales are currently in wide use in emergency department and critical care settings worldwide; however, only the CDS has been externally validated against the established criterion standard of percent weight change in a North American population [14].

**Table 1:** Clinical dehydration scale (CDS).

Characteristic	0	1	2
General appearance	Normal	Thirsty, restless, or lethargic, but irritable when touched	Drowsy, limp, cold, sweaty and/or comatose
Eyes	Normal	Slightly sunken	Very Sunken
Mucous membranes	Moist	“Sticky”	Dry
Tears	Tears	Decreased Tears	Absent Tears

Scoring: 0: no dehydration < 3%, 1-4: some dehydration ≥ 3% - 6%, 5-8: moderate dehydration ≥ 6%.

**Table 2:** World health organization scale.

	A	B	C
Look at condition	Well, alert	Restless, irritable	Lethargic or unconscious
Eyes	Normal	Sunken	Sunken
Thirst	Drinks normally, not thirsty	Thirsty, drinks eagerly	Drinks poorly or not able to drink
Feel: Skin pinch	Goes back quickly	Goes back slowly	Goes back very slowly

Scoring: Fewer than two signs from column B and C: no signs of dehydration, <5%; ≥ 2 signs in column B: moderate dehydration, 5-10%; ≥ 2 signs in column C: severe dehydration, > 10%.

**Table 3:** Gorelick 10-point scale.

Characteristic	No or minimal dehydration	Moderate to severe dehydration
General Appearance	Alert	Restless, lethargic, unconscious
Capillary refill	Normal	Prolonged or minimal
Tears	Present	Absent
Mucous Membranes	Moist	Dry, very dry
Eyes	Normal	Sunken, deeply sunken
Breathing	Present	Deep, deep and rapid
Quality of pulses	Normal	Thready, weak or impalpable
Skin elasticity	Instant recoil	Recoil slowly; recoil > 2 seconds

In this study, we investigate the accuracy of three popular clinical scales: the WHO scale, the Gorelick scale, and the Clinical Dehydration Scale (CDS).

## Methods

### Study Design

This was a prospective, non-consecutive cohort study of children between June 2011 and February 2013. The data in this study was collected as part of the Bedside Ultrasound to Detect Dehydration in Youth (BUDDY) study, which also investigated the accuracy of ultrasound for detecting significant dehydration in children. This study was approved by the Human Subjects Internal Review Board at Rhode Island Hospital in Providence, Rhode Island. Written informed consent was obtained by each subject's parent or legal guardian.

### Setting and Participants

Enrollment took place in the emergency department (ED) of Hasbro Children's Hospital, a regional pediatric referral hospital with an annual census of approximately 50,000 pediatric ED visits.

All children less than or equal to 18 years of age presenting with a chief complaint of vomiting and/or diarrhea, or suspicion of dehydration by an attending pediatric emergency physician were eligible for enrollment. Eligible patients whose initial order sets included plans for intravenous fluids were approached for study consent, and those only receiving oral fluids were not included in the study. Exclusion criteria included positive pressure ventilation, significant traumatic injury, large volume fluid administration prior to enrollment, surgical abdomen, and known congenital cardiac disease. Only English speaking patients were enrolled due to limited translator availability.

### Patient Assessment

Trained research assistants screened all patients presenting to the emergency department during study hours. After identifying a patient for enrollment, research assistants obtained written informed consent from all parents or legal guardians and verbal consent from the child, depending on their literacy.

### Calculation of Percentage of Dehydration

Research assistants weighed each child prior to intravenous fluid administration and again at the completion of ED resuscitation. Children admitted to the inpatient service for further hydration were weighed again prior to hospital discharge. All weights were performed without the child wearing clothes and with the same calibrated study scale (Seca Iena 354 for infants and Seca 813 Robusta for children able to stand, Seca, Handover, MD). The final weight was recorded as the weight upon discharge from the ED or from the hospital for admitted patients. The initial weight was that obtained upon enrollment. The percent dehydration was determined using the following formula: (final weight-initial weight)/final weight x 100%. Subjects with a percentage weight change of 5% or more were considered to be significantly dehydrated based on standards in the pediatric literature [5], [10], [15]-[16].

### Data Collection

After enrollment, the treating pediatric emergency medicine attending physician, who had examined the patient, recorded their overall gestalt of the severity of dehydration. Physician gestalt for our study is defined as the initial clinical impression of the treating physician for percent dehydration on a data sheet using a 1-10 scale. After recording their overall gestalt, the treating physician subsequently documented the subject's historical features and physical exam findings consistent with each clinical score (Tables 1, 2, and 3). The scores were not listed on the data sheet, only the components of the scores. Afterward, a research assistant documented the patient's vital signs, weight, clinical score and volume of fluid administered. None of the treating pediatric emergency medicine attending physicians were involved in the study apart from recording their clinical impression and the subject's historical features and physical exam findings as above. The treating physician had complete autonomy over the clinical decisions regarding management of the subjects.

### Outcomes

Our primary outcome was to assess the accuracy of the CDS, Gorelick and WHO scales for predicting the severity of dehydration in children relative to the criterion standard of percent weight change [5], [8]-[11], [13], [16]. Our secondary outcome was to assess the accuracy of overall physician gestalt compared to the same criterion standard of percent weight change with rehydration.

### Statistical Analysis

First, we calculated basic population demographics using descriptive statistics. We then constructed receiver operating characteristic (ROC) curves to evaluate the accuracy of the CDS, WHO scale, Gorelick Scale and physician gestalt compared to our criterion standard. We also calculated the sensitivity, specificity, likelihood ratio positive (LR+) and likelihood ratio negative (LR-) for each scale and physician gestalt [17]. We calculated these test characteristics using the best cut off points for the CDS and Gorelick scale that correlated with significant dehydration, defined as ≥ 5% weight change (≥2 for the CDS, and ≥2 for the Gorelick scale). We did not use a priori cut points originally derived in the CDS and Gorelick studies because one goal of our study was to find cut points on the ROC curve that provided the most clinically useful sensitivity and specificity in a United States hospital population. For the WHO scale we used the predefined cut off point of greater than or equal to 2, including both column B and C from the scale [12].

In addition, we conducted a sub-group analysis to evaluate the performance of each clinical scale based on the subject age for which they scales were originally designed, under 3 years of age for the CDS and under 5 years of age for the WHO and Gorelick scales [10]-[12].

Statistical analyses were performed using Stata version 11.0 (StataCorp LP, College Station, TX).

### Sample Size

The sample size was originally calculated based on the inferior vena cava (IVC) ultrasound component of the study. Using standard algorithms from the literature, an area under the ROC curve (AUC) of .756 for the performance of IVC ultrasound as a predictor of dehydration based on data previously obtained, a type I error rate of .05, a type II error rate of .20, and the proportion of children with significant (>5%) dehydration in prior studies conducted in North American hospitals, we determined we would need to enroll at least 112 children to be sure that the 95% confidence intervals for our ROC curve did not cross the reference line (or null hypothesis) [10]-[11], [18]-[19]. Significant dehydration was defined as moderate (5-10%) and severe (>10%) dehydration combined.

## Results

### Demographics

We approached 209 patients for potential enrollment, 61 declined to consent. Of the 61 children declining to consent, 35 were male, 26 were female and the median age was 3 years (range 1 week – 17 years). Of the 148 remaining patients, 35 withdrew from the study or were excluded prior to completion of data collection, leaving 113 children for analysis. Of the 35 children not included in the final analysis 17 were male, 18 were female and the median age was 4 years (range 1 month – 17 years). Of the 113 enrolled children, 39 were admitted and 74 were discharged from the ED with 58 male and 55 female. Median age for the patients enrolled was 6 years (range 1 month – 18 years). Twenty-nine children were under 3 years of age and 49 were under 5 years of age. Six children were under 6 months of age and 17 were under 2 years of age.

### Outcomes

Volumes of fluid resuscitation were determined clinically by the treating clinician and ranged from less than 20 cc/kg in 34% of patients, greater than 20 cc/kg but less than 40 cc/kg in 52% of patients, and greater than 40 cc/kg in 14% of patients. The average percent weight change with rehydration was 2.8%. Twelve patients (10.6%) had significant dehydration, defined as a percentage weight change greater than 5% with rehydration.

### Accuracy of Clinical Scales

**Figure 1:**
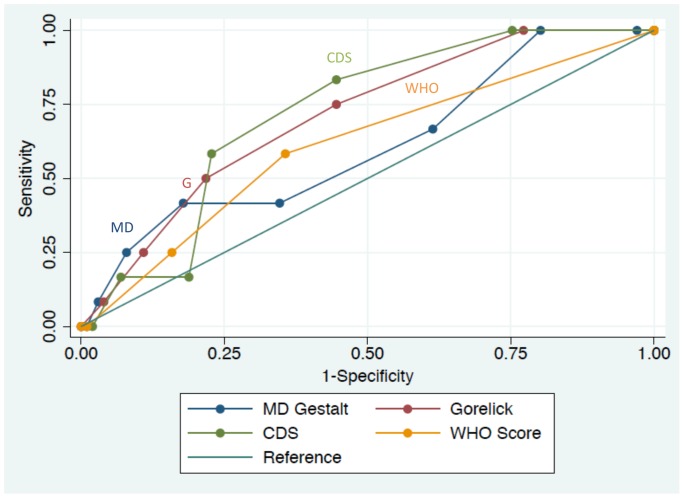
Receiver Operating Characteristic Curves. Abbreviations: CDS, Clinical Dehydration Scale; WHO, World Health Organization; G, Gorelick; MD, Physician.

Test characteristics for the three clinical scales and physician gestalt are shown in Table 4 and comparison of the ROC curves for each scale and gestalt are demonstrated in Figure 1. The CDS and the Gorelick scale both had an area under the ROC curve (AUC) statistically different from the reference line, and therefore, were both statistically associated predictors of the severity of dehydration. The AUCs for the CDS and Gorelick scale were 0.72 (95% CI 0.60, 0.84) and 0.71 (95% CI 0.57, 0.85) respectively. The WHO scale was not a significant predictor of the severity of dehydration in the overall study population. The AUC for the WHO scale was 0.61 (95% CI 0.45, 0.77).

**Table 4:** Test characteristics.

Technique (Cut Point)*	AUC (95% CI)	SN (95% CI)	SP (95% CI)	LR+ (95% CI)	LR- (95% CI)
**CDS (2)**	0.72 (0.60, 0.84)	83% (52%, 98%)	55% (45%, 65%)	1.87 (1.34, 2.61)	0.30 (0.08, 1.08)
**Gorelick (2)**	0.71 (0.57, 0.85)	75% (43%, 95%)	55% (45%, 65%)	1.68 (1.14, 2.49)	0.45 (0.17. 1.22)
**WHO (2)**	0.61 (0.45, 0.77)	25% (5%, 57%)	84% (76%, 91%)	1.58 (0.54, 4.64)	0.89 (0.64, 1.25)
**Physician Gestalt (5)**	0.61 (0.44, 0.78)	42% (15%, 72%)	65% (55%, 75%)	1.20 (0.59, 2.47)	0.89 (0.54, 1.47)

*The cut point refers to the position on the receiver operating characteristic curve that corresponds to the best test characteristics for the Clinical Dehydration Scale (CDS), Gorelick scale, and physician gestalt respectively. The World Health Organization (WHO) scale test characteristics are based on a predefined cut point. Abbreviations: AUC, area under the receiver operating characteristic curve; SN, sensitivity; SP, specificity; LR+, likelihood ratio positive; LR-, likelihood ratio negative.

At its best cut off point of 2 or more, the CDS had a sensitivity of 83%, a specificity of 55%, a likelihood ratio positive (LR+) of 1.87, and a likelihood ratio negative (LR-) of 0.30 for predicting significant dehydration. At its best cut off point of 2 or more, the Gorelick scale had a sensitivity of 75%, a specificity of 55%, a LR+ 1.68, and a LR- 0.45 for predicting significant dehydration. At its predefined cut off point of 2 or more, the WHO scale had a sensitivity of 25%, a specificity of 84%, LR+ of 1.58, and LR- of 0.89 for predicting significant dehydration.

### Subgroup Analysis by Age of Child

When we analyzed the clinical scales according to the specific age ranges for which the scales were originally defined, they were not significant predictors of the severity of dehydration. The AUC for the CDS, when limited to children less than 3 years of age, was 0.66 (95% CI 0.46, 0.86), while the AUC for the Gorelick scale, when limited to children less than 5 years of age, was 0.61 (95% CI 0.43, 0.80). The AUC for the WHO scale, when limited to children less than 5 years of age, was 0.59 (95% CI 0.41, 0.78). The test characteristics for this subgroup analysis are shown in Table 5.

**Table 5:** Subgroup analysis by age of child from which each scale was derived.

Age in Years	Number of Children (% of Enrolled)	CDS AUC (95% CI)	Gorelick AUC (95% CI)	WHO AUC (95% CI)
**< 3**	29 (25.7%)	0.66 (0.46, 0.86)		
**< 5**	49 (43.4%)		0.61 (0.45, 0.80)	0.59 (0.41, 0.78)

Abbreviations: CDS, Clinical Dehydration Scale; AUC, area under the receiver operating characteristic curve; CI, confidence interval.

### Physician Gestalt

Physician gestalt did not have an area under the curve statistically different from the reference line. The AUC for physician gestalt was 0.61 (0.44, 0.78) with sensitivity of 42% and specificity of 65%, likelihood ratio positive 1.20 and a likelihood ratio negative of 0.89 for predicting significant dehydration based on the cut-point of 5 or more.

## Discussion

Acute dehydration in pediatric patients is a common and potentially life-threatening condition encountered frequently in both primary care and emergency medicine practice. Unfortunately, individual symptoms and physical exam findings are often unhelpful in discerning the degree of dehydration in the pediatric patient, which is important for clinical decision-making [5]. A criterion standard used commonly in dehydration research for determining the severity of dehydration in pediatric patients is the patient's percent weight change, generally defined as the percent difference between the pre-illness weight and the acute-illness weight, or alternatively as the percent difference between the acute illness weight and the post-rehydration weight [5], [8]-[11], [13], [20]. However, this is not a useful method in clinical practice, as the patient's pre-illness weight is most often not available to providers at the time of presentation, when they must make a rapid decision as to whether or not the child is severely dehydrated enough to necessitate intravenous fluids [7], [10].

Historically, physicians have estimated the degree of dehydration in children based on clinical gestalt. This leads to variability amongst providers and has been shown not to correlate well with dehydration based on the gold standard [9]. Because of this, several clinical dehydration scores have been developed in order to aid in the diagnosis and management. We chose to study the CDS, Gorelick and WHO scale because of their worldwide use and prominence in the pediatric literature [10]-[15], [21]-[22].

Several previous studies have assessed the accuracy of clinical scales for predicting dehydration in children with diarrhea using the criterion standard of percent weight change with rehydration. Gorelick, et al. assessed the accuracy of a 10-point clinical scale in children with diarrhea presenting to a single pediatric referral hospital in Philadelphia, while Vega, et al. assessed the accuracy of a similar 9-point scale in children presenting to an academic medical center in New York [8], [10]. Gorelick, et al. found their 10-point scale to have a sensitivity of 82% and specificity of 90% for predicting severe dehydration in children when assessed by an experienced emergency nurse, while Vega, et al. found their 9-point scale to have a sensitivity of 70% and specificity of 84% for predicting severe dehydration when performed by an emergency physician. The Clinical Dehydration Scale was initially derived in a population of children presenting with diarrhea to a Canadian pediatric referral hospital and found to be a significant predictor of moderate-severe dehydration in that same population of children [9], [23]. Additional studies have found the Clinical Dehydration Scale to be a significant predictor of emergency department length of stay, treatment with intravenous fluids, and hospitalization, but not severity of dehydration [14], [15].

To our knowledge, the only prior study to assess the accuracy of the WHO scale against an established criterion standard was a small study conducted in three hospitals in Rwanda [24]. The authors of this study did not find the WHO severe dehydration scale to be an accurate predictor of severe dehydration in children, though it had limited power due to its small sample size. In addition, they did not find the Gorelick scale or the Clinical Dehydration Scale to be accurate predictors of severe dehydration when compared with the criterion standard of percent weight change. However, neither of these scales has been externally validated in a North American population from which they were originally derived.

In our study, the CDS and the Gorelick scale were both statistically significant but poor predictors of the severity of dehydration in our overall study population. However, they were not statistically significant predictors when limited to the age groups for which they were originally derived, possibly due to our small overall study numbers in those age groups. The WHO scale was no better than chance at predicting the severity of dehydration in either our overall study population or when limited to children age 1-60 months. In addition, similar to what has been demonstrated in the literature previously, physician gestalt was a poor predictor of the severity of dehydration. As such, while some clinical scales may be moderately useful and most physicians rely on their clinical impression for assessing the severity of dehydration in the emergency department the development of a better tool for assessing pediatric dehydration is needed.

### Limitations

This was a prospective, non-consecutive cohort study; children were enrolled based on research assistant availability. Furthermore, the data in this manuscript was collected as part of a broader study of dehydration in youth, and was originally powered based on the expected performance of IVC ultrasound as a predictor of dehydration. As such, the study was not specifically powered to demonstrate the superiority of any one clinical scale over another, to assess the accuracy of the scales only for the subgroup age ranges that they were originally designed for, or to show that any one scale performed better than physician gestalt. In addition, we excluded patients who had already received a significant volume of intravenous fluids, which likely includes the most acutely ill children receiving an immediate intravenous line and fluids on arrival in dedicated resuscitation rooms.

When calculating the percent weight change we used the final, post-rehydration weight as a surrogate marker for the pre-illness weight. As such, we assume the ED discharge weight and the hospital discharge weight reflect the pre-illness weight. This method was used in the derivation study of the CDS scale [11], [13]. In the derivation of the Gorelick scale, serial weights were measured until a stable weight, defined as a difference <2% between the last 2 weights, was achieved [10]. Furthermore, while recent research has found post-rehydration weight correlates almost perfectly with pre-illness weight, post-rehydration weight does tend to underestimate pre-illness weight by about 2% [23]. It is also theoretically possible that children received an unnecessarily large amount of fluids making their post-rehydration weight a reflection of fluid over load rather than true baseline weight of the patient.

Finally, we did not assess inter-rater reliability in this study. It has been shown that there may be significant variability amongst the scores clinicians assign patients when using a clinical dehydration score [25].

## Conclusion

This is the first study to externally validate the CDS and Gorelick scales as fair predictors of dehydration in children with diarrhea or vomiting based on the criterion standard of percent weight change with rehydration in a North American cohort. Neither scale remained a helpful predictor when limited to the younger populations of children for which they were originally intended, however this may have been due to our small sample size. Neither the WHO scale nor physician gestalt was found to be a helpful predictor of dehydration in our study cohort. Further research should focus on developing new tools to assess the severity of dehydration and fluid responsiveness in children with diarrhea and vomiting.
